# Differential Preincubation Effects of Nicardipine on OATP1B1- and OATP1B3-Mediated Transport in the Presence and Absence of Protein: Implications in Assessing OATP1B1- and OATP1B3-Mediated Drug–Drug Interactions

**DOI:** 10.3390/pharmaceutics15031020

**Published:** 2023-03-22

**Authors:** Ruhul Kayesh, Vishakha Tambe, Chao Xu, Wei Yue

**Affiliations:** 1 Department of Pharmaceutical Sciences, University of Oklahoma Health Sciences Center, Oklahoma City, OK 73117, USA; 2 Department of Biostatistics and Epidemiology, University of Oklahoma Health Sciences Center, Oklahoma City, OK 73104, USA

**Keywords:** drug–drug interactions, OATP, pharmacokinetics, drug transporters

## Abstract

Impaired transport activity of hepatic OATP1B1 and OATP1B3 due to drug–drug interactions (DDIs) often leads to increased systemic exposure to substrate drugs (e.g., lipid-lowering statins). Since dyslipidemia and hypertension frequently coexist, statins are often concurrently used with antihypertensives, including calcium channel blockers (CCBs). OATP1B1/1B3-related DDIs in humans have been reported for several CCBs. To date, the OATP1B1/1B3-mediated DDI potential of CCB nicardipine has not been assessed. The current study was designed to assess the OATP1B1- and OATP1B3-mediated DDI potential of nicardipine using the R-value model, following the US-FDA guidance. IC_50_ values of nicardipine against OATP1B1 and OATP1B3 were determined in transporter-overexpressing human embryonic kidney 293 cells using [^3^H]-estradiol 17β-D-glucuronide and [^3^H]-cholecystokinin-8 as substrates, respectively, with or without nicardipine-preincubation in protein-free Hanks’ Balanced Salt Solution (HBSS) or in fetal bovine serum (FBS)-containing culture medium. Preincubation with nicardipine for 30 min in protein-free HBSS buffer produced lower IC_50_ and higher R-values for both OATP1B1 and OATP1B3 compared to in FBS-containing medium, yielding IC_50_ values of 0.98 and 1.63 µM and R-values of 1.4 and 1.3 for OATP1B1 and OATP1B3, respectively. The R-values were higher than the US-FDA cut-off value of 1.1, supporting that nicardipine has the potential to cause OATP1B1/3-mediated DDIs. Current studies provide insight into the consideration of optimal preincubation conditions when assessing the OATP1B1/3-mediated DDIs in vitro.

## 1. Introduction

Organic anion transporter polypeptide (OATP) 1B1 and OATP1B3 are basolateral uptake transport proteins in human hepatocytes. Many clinically used drugs are substrates of OATP1B1 and OATP1B3, including widely prescribed lipid-lowering medicines [1]. Reduced transport activity of OATP1B1 and OATP1B3 due to drug–drug interactions (DDIs) and genetic variations have been reported to increase systemic exposure to statins and statin-induced myopathy [2,3]. 

Hypertension and dyslipidemia are two major risk factors for cardiovascular diseases that frequently coexist in patients [4]. Calcium channel blockers (CCBs) and statins are often prescribed for the treatment of hypertension [5] and dyslipidemia [6], respectively. Since simultaneous treatment of hypertension and dyslipidemia is beneficial to reduce the overall cardiovascular risk [4], concurrent administration of CCBs and statins is likely. For example, the single-pill therapy combining amlodipine, a CCB, and atorvastatin has proven effective in patients of diverse ethnicity, with respect to attaining recommended goals for blood pressure and lipids [7]. 

Of the current nine US-FDA-approved CCBs (amlodipine, felodipine, isradipine, nicardipine, nifedipine, nimodipine, nisoldipine, diltiazem, and verapamil) and the one withdrawn CCB (mibefradil), three, namely nisoldipine, verapamil, and mibefradil, have been reported to increase the area under the plasma concentration–time profile (AUC) of several OATP1B1 and/or OATP1B3 drug-substrates, including statins and telmisartan in humans [8,9,10]. This relatively high chance of potential DDIs (i.e., 3 out of 10 CCBs) against OATP1B1/3 substrates underscores the need to assess the OATP1B1 and OATP1B3-mediated DDI potential of those other CCBs, for which inhibition potency against OATP1B1 and OATP1B3 has not been characterized in vitro. 

Recently, time-dependent inhibition has been reported for some OATP1B1/3 inhibitors [11,12,13,14]. In these instances, preincubation with some OATP1B1/3 inhibitors led to reduced IC_50_ values against OATP1B1 and OATP1B3 and increased R-values when assessing OATP1B1- and OATP1B3-mediated DDIs. An inhibitor preincubation step is currently recommended in the US-FDA final guidance to mitigate false negative prediction of OATP1B1- and OATP1B3-mediated DDIs [15]. In addition to OATP1B1 and OATP1B3, preincubation with other transporters, such as organic cation transporters, has also been reported [16]. For inhibitors with reported preincubation effects on OATP1B1- and OATP1B3-mediated transport, the preincubation step in some studies was conducted in protein-free buffers, such as Hanks’ Balanced Salt Solution (HBSS) [12,13,14], or serum-free culture medium [11,13,17], while some were conducted in protein-containing cell culture medium supplemented with fetal bovine serum (FBS) in transporter-expressing cell lines [18]. To the best of our knowledge, the impact of serum in the preincubation buffer system, which is a source of proteins, on the inhibitor–preincubation effects on OATP1B1- and OATP1B3-mediated transport has not been reported. 

Nicardipine is a second-generation dihydropyridine CCB, which has shown excellent hypertension management in elderly patients [19,20]. Nicardipine was reported as an inhibitor of P-glycoprotein (P-gp) [21] and renal OATP4C1 [22]. Currently, only limited published literature exists regarding the effects of nicardipine on OATP1B1 and OATP1B3. Nicardipine (20 µM) in HBSS buffer was reported to inhibit OATP1B1-mediated transport by 65% [23]. However, the IC_50_ values of nicardipine and its effects on OATP1B1 and OATP1B3-mediated transport have not been reported. The aims of the current study are two-fold: (1) to determine the (IC_50_) of nicardipine and assess OATP1B1- and OATP1B3-mediated DDIs using the FDA-recommended R-value model [15]; (2) to determine the effects of preincubation of nicardipine, either in a protein-free buffer or in an FBS-containing medium, on OATP1B1- and OATP1B3-mediated transport. OATP1B1 and OATP1B3 overexpressing HEK293 cells were used for the current studies. Interestingly, differential effects of preincubation of nicardipine in protein-free buffer or FBS-containing medium were observed for both OATP1B1 and OATP1B3, with a greater effect observed in the protein-free buffer. 

## 2. Materials and Methods

### 2.1. Materials

[^3^H]-estradiol 17 β-D-glucuronide (E_2_17βG) (specific activity 37.2 Ci/mmol) and [^3^H]-cholecystokinin-8 (CCK-8) (specific activity 98.4 Ci/mmol) were purchased from Perkin Elmer Life Science (Waltham, MA, USA). Nicardipine was purchased from Santa Cruz Biotechnology (Dallas, TX, USA) Unlabeled CCK-8, E_2_17βG, dimethyl sulfoxide (DMSO), Dulbecco’s Modified Eagle Medium (DMEM), Hank’s Balanced Salt Solution (HBSS), antibiotic antimycotic solution, trypsin–EDTA solution, Triton X-100, and fetal bovine serum (FBS) were purchased from Sigma-Aldrich (St. Louis, MO, USA). Poly-L-lysine was purchased from Trevigen Inc. (Gaithersburg, MD, USA). Geneticin^®^ and HEPES were purchased from BD Biosciences (Bedford, MA, USA). Bio-Safe II liquid scintillation mixture was purchased from Research Products International (Mt. Prospect, IL, USA). 

### 2.2. Cell Culture

HEK293 stable cell lines expressing FLAG-tagged OATP1B1 (HEK293-FLAG-OATP1B1) [24] or FLAG-tagged OATP1B3 (HEK293-FLAG-OATP1B3) [25] have been published previously. Both HEK293 stable cell lines were cultured in DMEM culture media supplemented with 9% FBS (abbreviated as FBS–DMEM), antibiotic antimycotic, and 600 µg/mL G418 as published previously [24,25]. 

### 2.3. Transport Studies and IC_50_ Determination

HEK293-FLAG-OATP1B1 and HEK293-FLAG-OATP1B3 cells (less than 40 passages) were seeded in poly-L-lysine coated 24-well plates at a density of 1.5 × 10^5^ cells per well and allowed to grow for 48 h until confluent before the transport studies [26]. [^3^H]-E_2_17βG (1 µM, 2 min) and [^3^H]-CCK-8 (1 µM, 3 min) were used as substrates for OATP1B1 and OATP1B3 in HEK293-FLAG-OATP1B1 and HEK293-FLAG-OATP1B3 cells, respectively. Substrate accumulation time was at a linear uptake range and accumulation was determined in HBSS buffer containing 10 mM HEPES (pH 7.4), in the absence of protein, similar to that published previously [18,27]. E_2_17βG and CCK-8 concentrations were below the K_m_ values for OATP1B1 and OATP1B3, respectively [28], hence, the determined IC_50_ values approximate the K_i_ values. In the preincubation scenario, cells were preincubated with the vehicle control, 0.1% DMSO (*v*/*v*) or nicardipine at indicated concentrations ranging from 0.05 to 4 µM for designated times, in either the DMEM culture medium, which contains 9% FBS or in HBSS buffer supplemented with 10 mM HEPES (pH 7.4). After preincubation, cells were washed three times with HBSS buffer and incubated with HBSS buffer containing substrate, in the absence of vehicle control or inhibitor. In the coincubation scenario, substrate accumulation was determined in the presence of vehicle control or nicardipine without preincubation. In the pre plus coincubation scenario, substrate accumulation was determined in the presence of the vehicle control or nicardipine, following preincubation at the same concentration of the vehicle control or nicardipine described above and subsequently washed. At the end of substrate incubation, cells were washed three times with ice-cold HBSS buffer and lysed in PBS containing 0.5% Triton X-100. Liquid scintillation counting was determined and normalized to the protein concentration, after correcting for any nonspecific binding in the poly-L-lysine-coated blank plates, as published previously [18,27]. 

Estimation of the half maximal inhibitory concentration (IC_50_) values of nicardipine against OATP1B1 and OATP1B3 was the same as published previously using nonlinear regression (Equation (1)) in GraphPad Prism v.8.04 (GraphPad Software, La Jolla, CA, USA) [18].

(1) $$E=Bottom+\frac{Top-Bottom}{\left(1+\frac{C}{IC_{50}}\right)}$$

where E is the remaining substrate transport at a given inhibitor concentration (C). IC_50_ is the inhibitor concentration causing a response halfway between the maximally inhibited (bottom) response and the maximal (top) response. 

### 2.4. Prediction of OATP-Mediated DDIs Using the R-Value Model

The R-value, which represents the predicted AUC ratio of the substrate drug in the presence versus absence of an inhibitor drug, was calculated based on Equation (2), following the US-FDA final guidance [15] with the parameters summarized in Table 1.

(2) $$R=1+(f_{u,plasma}\times \frac{I_{In,\max}}{K_{i}})$$

A f_u, plasma_ of 0.05 is the unbound fraction of nicardipine in human plasma [29]. I_in, max_ is the estimated total maximum plasma concentration of nicardipine at the inlet to the liver and is estimated based on Equation (3), where I_max_ is the maximum plasma concentration (C_max_) of the inhibitor in the systemic circulation. Nicardipine doses were titrated, beginning with 20 mg, 3 times daily; doses in the range of 20 to 40 mg 3 times a day have been shown to be effective [29]. Steady-state C_max_ values following nicardipine 20, 30, and 40 mg doses, 3 times a day, are shown in Table 1. The f_a_ is the fraction absorbed. Nicardipine is completely absorbed following oral dosing [29], therefore, a f_a_ of 1 is used for nicardipine. F_g_ is the fraction that escaped gut metabolism (F_g_). The k_a_ is the absorption rate constant. Since values for F_g_ and k_a_ are unknown for nicardipine, values of F_g_ = 1 and k_a_ = 0.1/min were used as a worst-case estimate, according to the US-FDA guidance [15]. Q_h_ is the hepatic blood flow rate (1500 mL/min) [32]. R_B_ is the blood-to-plasma concentration ratio. In human blood, 12–18% of total nicardipine was present in erythrocytes [30]. Using a hematocrit of 0.45 as a default value, the R_B_ of nicardipine ranges from 0.63 to 0.67. R_B_ of 0.63 was used as a worst-case scenario.

(3) $$I_{in,\max}=I_{\max}+\frac{f_{a}F_{g}\times k_{a}\times Dose}{Q_{h}\times R_{B}}$$

As K_i_ approximates IC_50_, the R-value was determined as shown below:

(4) $$R=1+(f_{u,\mathrm{plasma}}\times \frac{I_{\mathrm{in},\max}}{{\mathrm{IC}}_{50}})$$

### 2.5. Statistical Analysis

**Figure 1:**
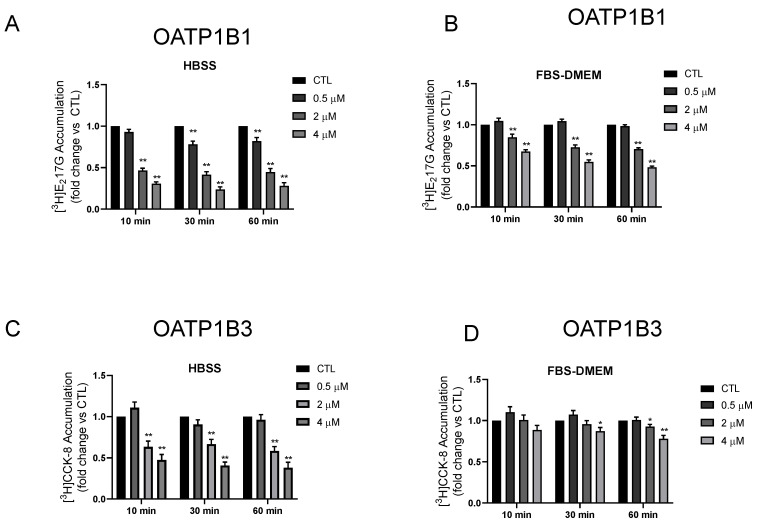
Effects of preincubation with nicardipine in HBSS buffer or FBS-containing medium on OATP1B1- and OATP1B3-mediated transport. Model-estimated fold changes and associated SE of the accumulation of [^3^H]-E_2_17βG (1 µM, 2 min) (**A**,**B**) and [^3^H]-CCK-8 (1 µM, 3 min) (**C**,**D**) vs. control (CTL) in HEK293-FLAG-OATP1B1 and HEK293-FLAG-OATP1B3 cells after preincubation with 0.5, 2, and 4 µM nicardipine for 10 min, 30 min, and 1 h in HBSS buffer or FBS-containing medium, as indicated in the legend. Substrate accumulation was determined in the absence of nicardipine after washing. Linear mixed-effects models were fit to the data, as described in the “Materials and Methods” (*n* = 3 in triplicate). * and ** indicate statistically significant differences vs. CTL with Bonferroni-adjusted *p*-values < 0.05 and 0.01, respectively.

For the statistical analysis shown in Figure 1, fold changes vs. control and associated standard errors (*SE*s) were estimated using linear mixed models with a random effect (experimental date) and a fixed group effect, allowing for group-specific variances, similar to those published previously [14,18]. In multiple comparisons, *p*-values were adjusted using Bonferroni’s method. A two-sided *p*-value of <0.05 defines statistical significance. SAS software (version 9.4, Cary, NC, USA) was used for statistical analyses. 

## 3. Results

### 3.1. Effects of Nicardipine-Preincubation on OATP1B1- and OATP1B3-Mediated Transport in HBSS Buffer, and FBS-Containing Medium

Well-characterized OATP1B1/3 inhibitor, Cyclosporine A (CsA), which has been reported to have preincubation-induced inhibitory effects on OATP1B1/3 [12,18] was used as a positive control. As shown in Figure S1, the preincubation IC_50_ values of CsA against OATP1B1 and OATP1B3, following 1 h CsA-preincubation (0–10 µM) in FBS–DMEM and subsequent washing, were 0.08 and 0.096 µM, respectively. These values were similar to those published previously (0.07 µM for OATP1B1 and 0.08 µM for OATP1B3) [18]. 

The preincubation effects of nicardipine (0.5–4 µM, 10 min–1 h) on OATP1B1- or OATP1B3-mediated transport were determined in two different preincubation media, HBSS buffer, and FBS-containing medium. As shown in Figure 1, the greatest inhibitory effects on OATP1B1 and OATP1B3 caused by nicardipine-preincubation were observed, in both, after nicardipine preincubation in the HBSS buffer. For OATP1B1, preincubation with 0.5 µM nicardipine significantly decreased OATP1B1-mediated transport after 30 min and 1 h preincubation in HBBS buffer by 0.78 ± 0.04 and 0.82 ± 0.04-fold of the control, respectively (Figure 1A); however, 0.5 µM nicardipine had no effect after a 10 min preincubation in HBSS buffer (Figure 1A), or at any preincubation duration in the FBS-containing medium (Figure 1B). Preincubation with nicardipine at 2 and 4 µM, in both the HBSS buffer (Figure 1A) and in the FBS-containing medium (Figure 1B), significantly reduced OATP1B1-mediated transport of [^3^H]-E_2_17G, at all preincubation time points, although a greater effect was seen in HBSS buffer than in the respective counterparts for FBS-containing medium (Figure 1A,B). 

For OATP1B3, preincubation with nicardipine at 0.5 µM did not significantly affect OATP1B3-mediated transport under any treatment condition (Figure 1C,D). However, in the HBSS buffer, preincubation with 2 or 4 µM nicardipine significantly decreased OATP1B3-mediated transport at all time points (10 min–1 h), with transport activity ranging from 0.41 ± 0.04 to 0.47 ± 0.03, and 0.24 ± 0.03 to 0.31 ± 0.02-fold of the control for 2 and 4 µM nicardipine, respectively (Figure 1C). In the FBS-containing medium, small, yet significant, reductions in OATP1B3-mediated transport were observed after preincubation with nicardipine at 4 µM for 30 min and at 2 or 4 µM for 1 h (Figure 1D). 

In the HBSS buffer, for both 2 or 4 µM nicardipine preincubations, the inhibitory effects were greater on both OATP1B1- and OATP1B3-mediated transports following 30 min and 1 h preincubations than after 10 min preincubation, yet inhibition was similar following 30 min and 1 h for both transporters for each nicardipine concentration. Hence, a 30 min preincubation timepoint was chosen in the subsequent studies to determine the effects of nicardipine preincubation on the IC_50_ values for OATP1B1 and OATP1B3. 

### 3.2. Effects of Nicardipine Preincubation on IC_50_ Values against OATP1B1 and OATP1B3 in HBSS and FBS-Containing Medium

**Figure 2:**
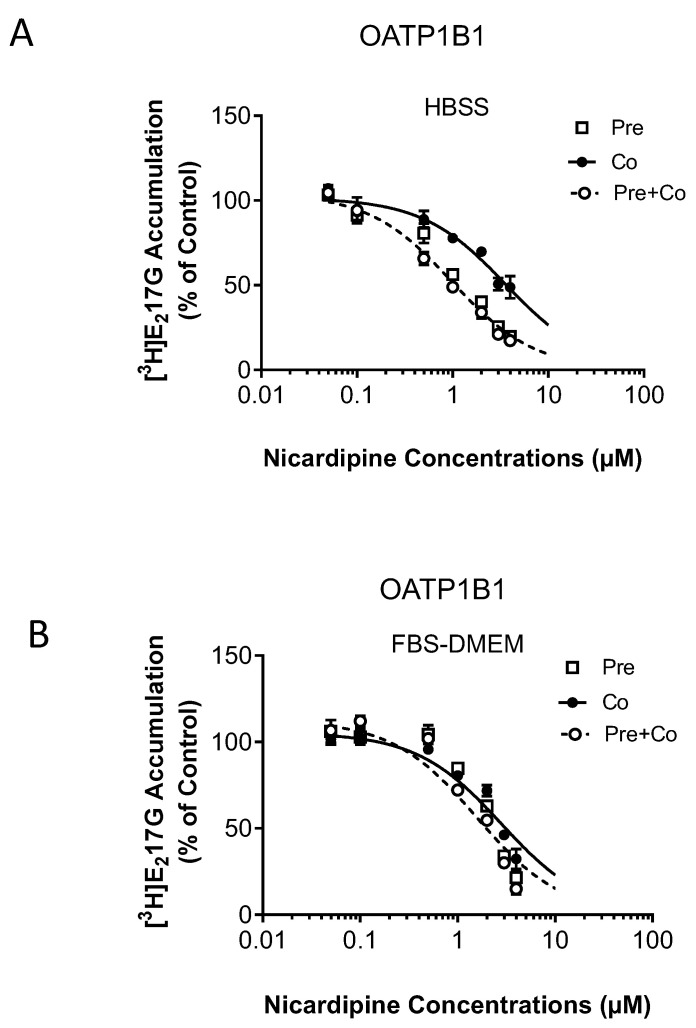
Effects of preincubation with nicardipine in HBSS buffer or FBS-containing medium on the IC_50_ value for OATP1B1. OATP1B1-mediated transport of [^3^H]-E_2_17βG is expressed as a percentage of vehicle control after 30 min preincubation in nicardipine-containing HBSS buffer (**A**) or FBS–DMEM (**B**) (open squares), coincubation (closed circles) and pre + coincubation (open circles) scenarios with nicardipine (0.05–4 µM) as the inhibitor are as described in the Materials and Methods. Data represent mean ± SEM (*n* = 3 in triplicate). The IC_50_ values were determined by fitting dose-response curves to the data using nonlinear regression analysis. Solid (coincubation) and dashed (pre + coincubation) lines represent the fitted lines.

**Figure 3:**
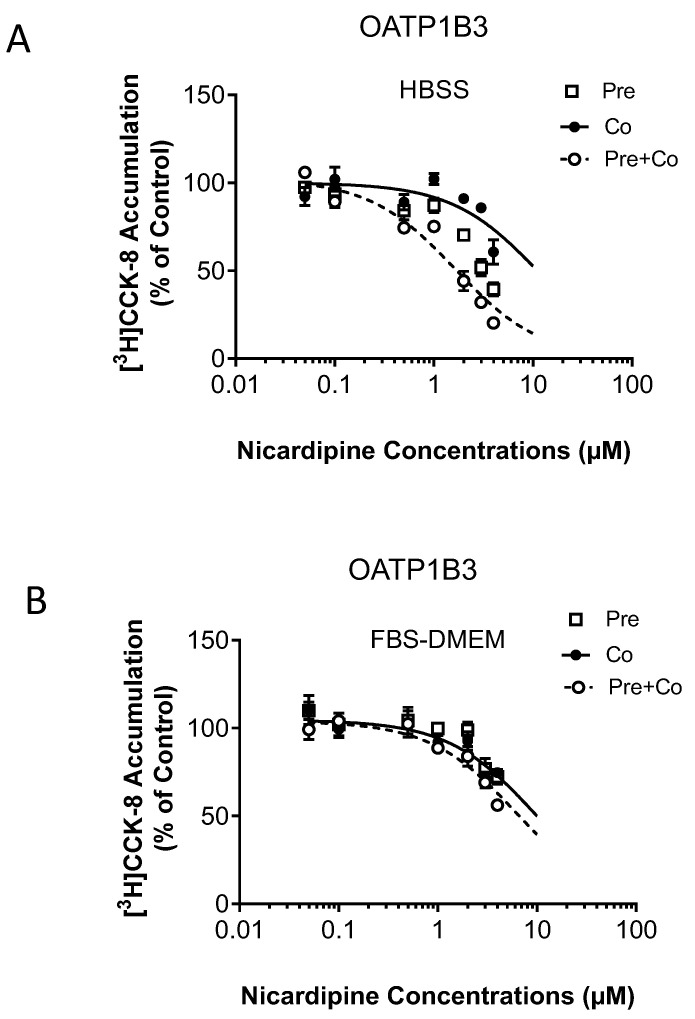
Effects of preincubation with nicardipine in HBSS buffer or FBS-containing medium on the IC_50_ values for OATP1B3. OATP1B3-mediated transport of [^3^H]-CCK-8 is expressed as a percentage of vehicle control after 30 min preincubation in nicardipine-containing HBSS buffer (**A**) or FBS–DMEM (**B**) (open squares), coincubation (closed circles) and pre + coincubation (open circles) scenarios with nicardipine (0.05–4 µM) as the inhibitor are as described in the Materials and Methods. Data represent mean ± SEM (*n* = 3 in triplicate). The IC_50_ values were determined by fitting dose-response curves to the data using nonlinear regression analysis. Solid (coincubation) and dashed (pre + coincubation) lines represent the fitted lines.

IC_50_ values for nicardipine against OATP1B1-mediated transport of [^3^H]-E_2_17βG (Figure 2) and OATP1B3-mediated transport of [^3^H]-CCK-8 (Figure 3) were determined by coincubation with either the vehicle control or nicardipine (up to 4 µM maximum aqueous soluble concentration) and either with (pre plus coincubation) or without (coincubation) 30 min nicardipine preincubation in HBSS buffer or FBS-containing medium. For comparison purposes, the preincubation data in Figure 1 and coincubation data in Figure 2 were replotted in Figure S2. 

The IC_50_ value of nicardipine against OATP1B1 was decreased by 3.6-fold (3.55 vs. 0.98 µM) and 1.8-fold (2.80 vs. 1.57 µM) after nicardipine preincubation in HBSS buffer and the FBS-containing medium, respectively (Figure 2 and Table 2). For OATP1B3, a 30 min preincubation followed by coincubation with nicardipine in HBSS buffer yielded an IC_50_ value of 1.63 µM. Notably, IC_50_ values for nicardipine against OATP1B3 could not be confidently estimated in other conditions, including with coincubation-only and pre plus coincubation in the FBS-containing medium, due to the less efficient inhibitions (Figure 3 and Table 2). 

### 3.3. Prediction of OATP-Mediated DDIs Using Static R-Value Model

The R-values for nicardipine against OATP1B1 and OATP1B3 at doses ranging from 20 to 40 mg are summarized in Table 2. For both OATP1B1 and OATP1B3, the R-values were higher in the pre plus coincubation condition than in the coincubation condition for each dose. In the pre plus coincubation condition, the IC_50_ values from inhibitor-preincubation in the HBSS buffer (pre+co-IC_50_,_HBSS_) were lower than those for the FBS–DMEM (pre+co-IC_50_,_FBS_), resulting in higher R-values at each dose for the HBSS buffer. 

Under the pre plus coincubation condition, for OATP1B1, the R-values determined from pre+co-IC_50_,_HBSS,OATP1B1_ were higher than the US-FDA recommended DDI cut-off value of 1.1, at all doses, ranging from 1.2 to 1.4, which supports the OATP1B1-mediated DDI potential of nicardipine, even at a low dose of 20 mg. For OATP1B3, the R-values were only higher than the DDI cut-off value of 1.1 when using the pre+co-IC_50_,_HBSS_ values at higher doses of 30 and 40 mg, suggesting a DDI potential against OATP1B3 at higher doses but not at the lowest dose of 20 mg. 

## 4. Discussion

The current study is the first to characterize the IC_50_ values of nicardipine against OATP1B1 and OATP1B3 and report that nicardipine has the potential to cause OATP1B1- and OATP1B3-mediated DDIs after following the current US-FDA recommended R-value model.

Interestingly, for the first time, the current study demonstrated differential inhibitor-preincubation effects in a protein-free HBSS buffer compared to an FBS-containing medium. Using the same preincubation time, the preincubation-induced inhibitory effects of nicardipine on OATP1B1- and OATP1B3-mediated transport appear to be concentration-dependent (Figure 1), with greater inhibitory effects observed at higher concentrations. A major component of FBS is bovine serum albumin (BSA) (~2.5 mg/mL in FBS [33]). The BSA (final concentration ~0.25% (*w*/*v*)) in the FBS–DMEM preincubation medium provides a source for protein binding. In addition to the DMEM medium, in the HBSS buffer, the nicardipine preincubation effects were also greater when preincubation was conducted in the absence of FBS, compared to those in the presence of FBS, for both OATP1B1 (Figure S3A and Figure 1A) and OATP1B3 (Figure S3B and Figure 1C). Since nicardipine is highly protein-bound (f_u_ = 0.05), the presence of BSA is anticipated to reduce the unbound concentrations of nicardipine and, therefore, may lead to a reduced inhibitory effect in the FBS-containing medium, compared to the protein-free HBSS buffer. More inhibitor drugs with a different f_u_ would need to be tested in this way in order to draw definitive conclusions. The current data, reported herein, show the complexity involved in determining the inhibition potency of OATP1B1 and OATP1B3 in the preincubation condition and underline the value in considering the proteins present in the preincubation medium when testing preincubation effects. 

Predicting transporter-mediated DDIs has continued to be challenging [34,35], where false negative predictions or large underestimations of DDIs have been reported [35,36]. Of note, all three reported DDIs of CCB against OATP1B1/3 drug substrates [8,9,10] were falsely predicted as negative [35,36]. Based on reported in vitro IC_50_ values for nisoldipine [36], verapamil [37], and mibefradil [36] against OATP1B1 and/or OATP1B3, these CCBs are not anticipated to cause clinical DDIs against OATP1B1/3 substrates [35,36]. However, during in vivo investigations in humans, the coadministration of nisoldipine significantly increased the AUC of telmisartan, a substrate of OATP1B3 [38], by 2.32-fold [9]. Coadministration of verapamil increased the AUC of pravastatin by 1.32-fold [8], while coadministration of mibefradil increased the AUC of pravastatin and atorvastatin by 1.32-fold and 4.43-fold, respectively [10] (Table 3). Pravastatin is a substrate of OATP1B1 [13], and atorvastatin is a substrate of both OATP1B1 [13] and OATP1B3 [23]. Pravastatin is a metabolically stable statin [39], while atorvastatin it is a substrate of CYP3A4 [40]. The DDI of mibefradil against atorvastatin has been postulated to be due to CYP inhibition [10]. However, hepatic uptake processes via OATPs have been shown to contribute predominantly to the hepatic elimination of atorvastatin at a subtherapeutic dose [41]. Subjects with the OATP1B1 c. 521CC genotype, which has reduced transport activity, has an increased AUC for pravastatin and atorvastatin of 1.9- and 2.4-fold of the reference, respectively (reviewed in [42]). Thus, a potential role of hepatic OATP1B1/3 in the DDI of mibefradil against pravastatin and atorvastatin cannot be excluded. 

The reported IC_50_ values of nisoldipine, verapamil, and mibefradil against OATP1B1 and OATP1B3 were all determined without inhibitor preincubation [36,37]. In the current study, adding a 30 min nicardipine preincubation step led to reduced IC_50_- and increased R-values, compared to no preincubation step, against OATP1B1 and OATP1B3 (Table 2). The inhibitor preincubation step shifted the prediction of DDI from no DDI potential or DDI potential only at the highest dose of nicardipine for OATP1B1 to DDI potential even at the lower doses (Table 2). Substrate-dependent inhibition of OATP1B1 has been reported [26], where E_2_17βG has been recognized as a sensitive probe substrate of OATP1B1 in vitro. Recently, coproporphyrin I (CP-1), an endogenous substrate of OATP1B [43], has been reported as a promising endogenous biomarker with which to evaluate in vivo inhibition of OATP1B1 in humans [44]. The effects of nicardipine on OATP1B1/3-mediated transport in vitro are worth evaluating using CP-1 as the substrate for potential translational purposes. To the best of our knowledge, no in vivo studies on nicardipine against OATP1B1 and/or OATP1B3 drug substrates have been reported. The current prediction performance using the R-value model warrants further verification using a physiologically based pharmacokinetic model (PBPK) and a clinical DDI study of nicardipine against OATP1B1/3 substrate, such as with statins. 

The CCB amlodipine has been predicted to have no DDI potential based on the IC_50_ values against OATP1B1, determined without an inhibitor-preincubation step [45]. Isradipine and nifedipine, both at 20 µM, have been reported to inhibit OATP1B1-mediated transport by 47.5% [23] and >50% [23], respectively. Diltiazem does not inhibit OATP1B1, even at 100 µM [45]. Based on our report, herein, on the potential DDI of nicardipine against OATP1B1 and OATP1B3 and the previously reported apparent high chance of CCBs causing DDIs with OATP substrate drugs [35,36], the OATP1B1- and OATP1B3-mediated DDI potential of other CCBs warrants further evaluation, following the addition of an inhibitor preincubation step. 

In conclusion, the present study reports that preincubation with nicardipine in a protein-free HBSS buffer elicits the lowest IC_50_ values against OATP1B1 and OATP1B3, compared to coincubation-only or preincubation in FBS-containing medium. The current study predicts that nicardipine has the potential to cause hepatic OATP1B-mediated DDIs. Adding an inhibitor preincubation step may help to mitigate the false-negative predictions that are often associated with CCBs and warrants further studies with other CCBs alongside verification in clinical studies.

## Figures and Tables

**Table 1:** Summary of nicardipine parameters for R-value determination.

Parameters	Values
Molecular Weight (g/mol)	515.99 [29]
Dose (mg)	20, 30, 40 [29]
C_max, plasma_ (μM)	0.07, 0.17, 0.26 [29]
Blood to plasma concentration ratio (C_b/p_)	0.63 [30]
k_a_ (Absorption rate constant: min^−1^)	0.1
f_u_ (Fraction unbound in the plasma)	0.05 [29]
f_a_ × F_g_	1 [31]
I_in, max_ (Estimated based on Equation (3): μM)	8.46

**Table 2:** IC_50_ values for nicardipine against OATP1B1 and OATP1B3 and predicted AUC ratios (R) of OATP1B1- and OATP1B3-mediated DDIs using the R-value model.

Transporter and Preincubation Buffer	Co-Incubation				Pre + Co-Incubation			
	IC_50_ (µM)	R (20 mg)	R (30 mg)	R (40 mg)	IC_50_ (µM)	R (20 mg)	R (30 mg)	R (40 mg)
**OATP1B1**								
HBSS	3.55 ± 2.46	1.1	1.1	1.1	0.98 ± 0.26	1.2	1.3	1.4
FBS-DMEM	2.80 ± 2.44	1.1	1.1	1.2	1.57 ± 1.40	1.1	1.2	1.3
**OATP1B3**								
HBSS	11.1 ± 60.0	1.0	1.0	1.0	1.63 ± 1.05	1.1	1.2	1.3
FBS-DMEM	9.17 ± 23.1	1.0	1.1	1.1	6.13 ± 8.10	1.1	1.1	1.1

**Table 3:** In vivo DDI of CCBs against OATP1B1/3 drug substrates.

OATP1B1/1B3 Substrates	CCBs	AUC Ratio	Reference
Telmisartan	Nisoldipine	2.32	[9]
Pravastatin	Verapamil	1.32	[8]
Simvastatin	Verapamil	4.22	[8]
Simvastatin Acid	Verapamil	4.25	[8]
Pravastatin	Mibefradil	1.32	[10]
Atorvastatin	Mibefradil	4.43	[8]

## Data Availability

Data are contained within the article.

## Supplementary Materials

The following supporting information can be downloaded at: https://www.mdpi.com/article/10.3390/pharmaceutics15031020/s1. Figure S1: Preincubation IC_50_ values of CsA against OATP1B1 and OATP1B3, Figure S2: Comparison of effects of 10-min pre-incubation and co-incubation with nicardipine, Figure S3: Effects of preincubation with nicardipine in FBS-containing HBSS on OATP1B1- and OATP1B3-mediated transport.

## Abbreviations

AUC: area under the plasma concentration–time curve; DDI: drug–drug interaction; DMEM: Dulbecco’s Modified Eagle Medium; DMSO: dimethyl sulfoxide; FBS: fetal bovine serum; HBSS: Hanks’ Balanced Salt Solution; IC_50_: inhibitor concentration producing 50% inhibition; K_i_: inhibition constant; OATP: organic anion-transporting polypeptide.
