# Supplementary material for: Differential Preincubation Effects of Nicardipine on OATP1B1- and OATP1B3-Mediated Transport in the Presence and Absence of Protein: Implications in Assessing OATP1B1- and OATP1B3-Mediated Drug–Drug Interactions
Source: Pharmaceutics. 2023 Mar 22;15(3):1020. doi: 10.3390/pharmaceutics15031020 (PMC10052025; doi:10.3390/pharmaceutics15031020)
Supplement: Supplementary file 1 [file pharmaceutics-15-01020-s001.zip › pharmaceutics-2121405-supplementary.pdf]

Supplemental Materials

Title:

Differential preincubation effects of nicardipine on OATP1B1- and OATP1B3-mediated transport in the presence and absence of protein: implications in assessing OATP1B1- and OATP1B3-mediated drug-drug interactions

Authors' names:

Ruhul Kayesh, Vishakha Tambe, Chao Xu and Wei Yue

Journal: Pharmaceutics

Year: 2023

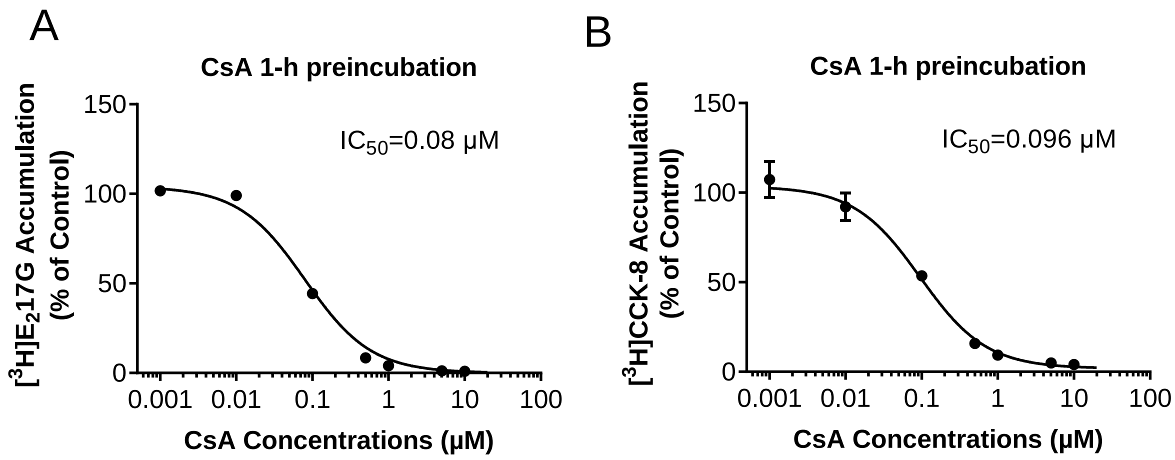

Figure S1. Preincubation IC_{50} values of CsA against OATP1B1 and OATP1B3. HEK293-FLAG-OATP1B1 and HEK293-FLAG-OATP1B3 cells were preincubation with CsA at indicated concentrations for 1 h in FBS-DMEM culture medium followed by washing. OATP1B1-mediated [^{3}H]$E_2$17βG accumulation (1 μM, 2 min) (A) and OATP1B3-mediated [^{3}H]CCK-8 (1 μM, 3 min) accumulation (B) were determined in the absence of CsA in protein-free HBSS (10 mM HEPES, pH 7.4). Data represents mean ± SD (n=1 in triplicate). Error bars in (A) are smaller than the symbol and appear invisible. The IC_{50} values were determined by fitting dose response curves to the data via nonlinear regression analysis same as described in the Material and Methods. Solid lines represent the fitted curves.

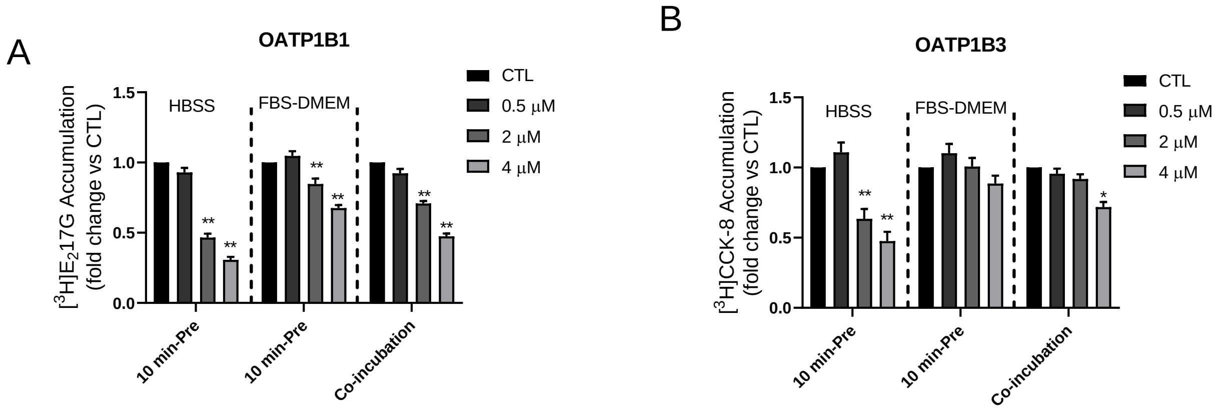

Figure S2. Comparison of effects of 10-min pre-incubation and co-incubation with nicardipine. The same 10 min-preincubation data in Fig. 1 and the same co-incubation data in Fig. 2 and Fig. 3 were replotted here for comparison purpose. Data represent model-estimated fold changes and associated SE vs. vehicle control treatment for both pre-incubation (N=3 for both OATP1B1 and OATP1B3) and co-incubation data (N=6), all in triplicate. Preincubation solution (HBSS or FBS-DMEM) are indicated. Linear mixed effects models were fit to the data as described in the “Materials and Methods”. * and ** indicates statistically significant differences vs. CTL with Bonferroni-adjusted p values <0.05 and 0.01, respectively.

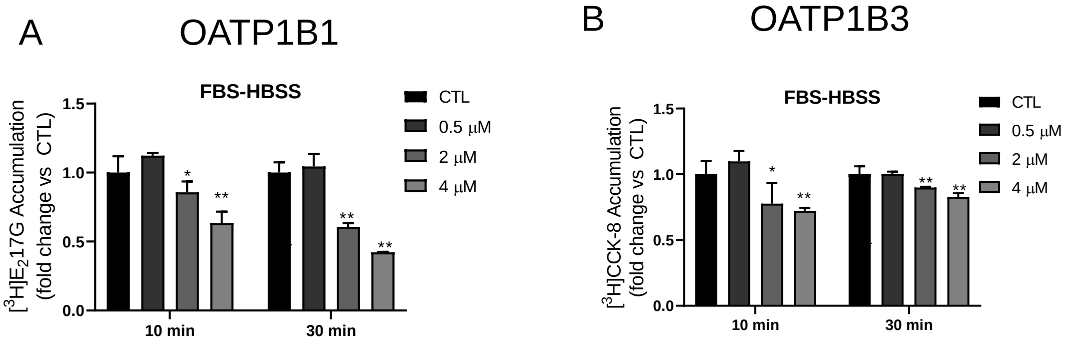

Figure S3. Effects of preincubation with nicardipine in FBS-containing HBSS on OATP1B1- and OATP1B3-mediated transport. (A) OATP1B1-mediated accumulation of [^{3}H]E217βG (1 μM, 2 min) and (B) OATP1B3-mediated accumulation of [^{3}H]CCK-8 (1 μM, 3 min) after 10 or 30 min preincubation with vehicle control (CTL) or nicardipine (0.5-4 μM). After preincubation and washing, substrate transport was determined in the absence of protein in HBSS (10 mM HEPES, pH 7.4) buffer. Data represent mean ± SD (n=1 in triplicate). * and ** indicates statistically significant differences vs. CTL by one way analysis of variance (ANOVA) with Bonferroni-adjusted p values <0.05 and 0.01, respectively.
